# Bisphosphonate-Related Osteonecrosis of the Jaw: A Review of the Literature

**DOI:** 10.1155/2014/192320

**Published:** 2014-04-28

**Authors:** Eder Alberto Sigua-Rodriguez, Renato da Costa Ribeiro, Ana Caroline Ramos de Brito, Natalia Alvarez-Pinzon, José Ricardo de Albergaria-Barbosa

**Affiliations:** ^1^Department of Oral and Maxillofacial Surgery, Piracicaba Dental School, P.O. Box 52, University of Campinas (UNICAMP), 13414-903 Piracicaba, SP, Brazil; ^2^Department of Dental Radiology, Piracicaba Dental School, P.O. Box 52, University of Campinas (UNICAMP), 13414-903 Piracicaba, SP, Brazil; ^3^Department of Prosthesis and Periodontology, Piracicaba Dental School, P.O. Box 52, University of Campinas (UNICAMP), 13414-903 Piracicaba, SP, Brazil

## Abstract

Bisphosphonates (BPs) are a class of drugs used to treat osteoporosis and malignant bone metastasis. BPs show high binding capacity to the bone matrix, especially in sites of active bone metabolism. The American Society for Bone and Mineral Research defines BRONJ as “an area of exposed bone in the maxillofacial region that has not healed within 8 weeks after identification by a healthcare provider in a patient who is receiving or has been exposed to a bisphosphonate and has not had radiation therapy to the craniofacial region.” Bisphosphonate-related osteonecrosis of the jaw (BRONJ) can adversely affect quality of life, as it may produce significant morbidity. The American Association of Oral and Maxillofacial Surgeons (AAOMS) considers as vitally important that information on BRONJ be disseminated to other dental and medical specialties. The purpose of this work is to offer a perspective on how dentists should manage patients on BPs, to show the benefits of accurately diagnosing BRONJ, and to present diagnostic aids and treatments strategies for the condition.

## 1. Introduction

Bisphosphonates (BPs) were first synthesized in 1865 in Germany [1]. Since then, BPs have been widely used in industry, in applications such as corrosion inhibition and fertilizers. As these drugs inhibit calcium carbonate precipitation, their use as blockers of bone resorption has been strongly advocated [1]. BPs such as alendronate, risedronate, ibandronate, and clodronate are used to treat several metabolic and oncologic pathologies that promote the destruction of the skeletal system.

BPs are divided into two main categories, that is, nonnitrogenated and nitrogenated [2]. Examples of nonnitrogenated BPs are etidronate and clodronate, while zoledronic acid, pamidronate, and ibandronate are nitrogenated BPs [3].

BPs, wrongly referred to as disphosphonates in the past, are compounds characterized by two C–P bonds. If both bonds are located in the same carbon atom, the compounds are considered germinal BPs, which are analogues of pyrophosphate containing an atom of oxygen replacing an atom of carbon. While hydrolysis easily dissociates pyrophosphates, BPs are resistant to that process. Thus, they have a long half-life—one of the main reasons for BPs accumulation in the bone matrix [4]. These drugs suppress osteoclast activity, reducing bone resorption and increasing bone density. Nowadays, their main medical use is the prevention and/or treatment of osteoporosis, osteopenia, multiple myeloma, malignant tumor metastases to the bone, and Paget's disease [5].

In patients treated with oral or intravenous BPs, bisphosphonate-related osteonecrosis of the jaw (BRONJ) has been and continues to be reported as a relatively rare, but potentially severe complication. It is characterized clinically as an area of exposed bone in the maxilla or the mandible that has failed to heal within a period of six to eight weeks in a patient currently or previously exposed to N-BPs who has not undergone radiation therapy in craniofacial region [6–8]. BRONJ progression is three-staged, which are identified based on clinical signs and symptoms. Recently, phase zero has been added in order to include high risk patients with no clinical evidence of necrotic bone, but with unspecific clinical signs and symptoms [9].

When administered orally, BPS absorption is low, in rates equal to or below 1% of the total dose [10]. When given intravenously, they are rapidly removed by the plasma and show a 40% renal excretion rate in the first 24 hours, without metabolization. While the half-life of BPs in the plasma is short, in bone it lasts for about 10 years [11]. Different groups of BPs may act through distinct mechanisms, but the final results are similar, that is, sharp decreases in osteoclastic activity and induction of apoptosis [12].

As BPs show great affinity for Ca^2+^ ions, mineralized bone matrix is a natural destination for these drugs. Their chemical structure provides resistance to enzymatic hydrolysis and allows BPs to bind avidly to the surface of hydroxyapatite crystals, creating a rapid and effective link between the drug and bone mineral surface [13]. Once deposited on bone surfaces, BPs promote osteoclastic apoptosis, hindering any subsequent osteoclast-mediated bone resorption [14].

Although the full mechanism of action of BPs is poorly understood, there are reports on their antiangiogenic properties as decreases in circulating levels of vascular endothelial growth factor have been observed [15]. The antiangiogenic effect of zoledronate (a 3rd generation BP) was demonstrated in rats, supporting its use in the treatment of malignant bone diseases, as well as in bone diseases with angiogenic components [15]. Oral BPs are chiefly used to treat osteoporosis and are not effective in treating malignant osteolytic lesions [16]. BPs may cause adverse reactions and most of them are related to the gastrointestinal system, such as nausea, vomiting, diarrhea, esophagitis with potential progress to esophageal ulcers; in addition, bone, muscle, and joint pain and allergic reactions are other possible adverse effects.

## 2. Bisphosphonate-Related Osteonecrosis of the Jaw (BRONJ) 

A new complication of interest to the dental profession has been recently referred to as BRONJ. It is a serious, albeit rare adverse reaction affecting jaw bones through an unknown mechanism, with potential to cause catastrophic tissue destruction [18].

According to AAOMS, BRONJ is defined as “necrotic bone exposed in maxillofacial region lasting for more than eight weeks in BPs-treated patients who have not undergone head and neck radiation therapy” [9].

The condition seems to be restricted to maxillomandibular complex, hence the name and the acronym BRONJ; there are no works reporting on similar lesions elsewhere in the body. An explanation for such fact would be the presence of teeth [6, 7], as they render the jaws as the only bones of the body that have an unimpeded connection with the exterior [18, 19]. In addition, teeth may suffer from periodontal disease, abscesses, endodontic injuries, and other lesions, conditions that require appropriate bone metabolism and blood supply to regain homeostasis [7]. Exposure to intravenous BPs for the management of malignancy remains the sole main risk factor for the development of BRONJ, as the prevalence in such patients ranges from 0.8 to 12%. Patients on oral BPs have a considerably lower risk to develop BRONJ when compared to cancer patients receiving intravenous BPs on a monthly basis. Based on data of the manufacturers of alendronate (MERCK laboratory), the risk of BRONJ in patients on oral treatment was calculated to be 0.7/100,000 person/year of exposure [9].

## 3. Staging 

In 2006, Ruggiero et al. [17], supported by their experience in diagnosing and managing 141 patients with BRONJ, implemented a staging system to group patients shown in Table 1.

In patients with clinically evident BRONJ, necrotic and infected bone is exposed to the oral environment, and erythema and edema of the surrounding soft tissue may be present [20]. In 25 to 40% of the cases, osteonecrosis appears in a spontaneous manner, without relation to any particular trauma or triggering condition [18, 19]. Spontaneous cases may be attributed to anatomic and physiologic traits, as they usually occur in the posterior region of the jaw where oral mucosa is thin. This is the most affected region, followed by the posterior maxilla, and mainly after dental extraction is performed [18].

In spontaneous cases, the most frequent initial symptom is an uncomfortable feeling in the mouth (paresthesia or burning sensation), with gradual changes in the mucosa, progressing to slow-healing ulcers. Pain may be intense and it is usually caused by necrotic bone infection by oral bacterial flora. These signs and symptoms may precede clinical evidence of osteonecrosis and it is essential to recognize them in order to take all possible preventive measures, since osteonecrosis is a progressive disorder that causes extensive exposition of jaw bones that may result in bone sequestra [21].

## 4. Diagnosis 

Diagnosis is very clear, directed by anamnesis, the history of oncologic pathology, and/or administration of BPs. Clinically evident lesions are confirmed through conventional radiographs showing radiopaque sequestrations, which are usually round with irregular peripheral radiolucencies [22].

Radiologic and nuclear medicine imaging may be valuable in recognizing and defining bone lesions in patients undergoing BPs therapy [21]. In the early phases of BRONJ, radiographic manifestations are not detected; however, as the disease progresses, osteonecrosis of the jaw may become readily identifiable in X-rays. When BRONJ is established, a poorly defined osteolytic area is seen along with cortical destruction, loss of cancellous trabeculation, and a decrease in bone density (similar to the radiological findings of osteomyelitis). Early osteonecrosis restricted to small areas of bone exposure (<1 cm) may be undetectable in panoramic radiographs; however, signs of bone destruction arising from this process may be recognized in computed tomography [23]. Computed tomography (CT) may allow a greater definition of the necrotic focuses and their relationship with neighboring anatomic structures, making it possible to quantify the status of bone sclerosis. However, CT may not be useful in staging asymptomatic patients [22].

According to Chiandussi et al. [21], scintigraphy exams may be useful in initial assessment of BRONJ patients. In some patients, there seemed to be a significant decrease or even complete absence of radioisotope intake, indicating low bone metabolism due to the absence of blood supply. However, these imaging resources are not able to show the difference between BRONJ and other causes of bone exposure in the jaw, such as osteoradionecrosis, osteomyelitis-related osteonecrosis of the jaw, or steroid-induced osteonecrosis [23]. Scintigraphy (Tc99-scan) is the most sensitive diagnostic strategy to identify edema and vascular changes and to locate bone necrosis even in the early stages of the disease. Nevertheless, this diagnostic technique has limitations: Tc99-scan is unable to distinguish BRONJ from metastatic processes [21, 24]. Biopsy of bone lesions must be carefully evaluated because the procedure itself may damage the bone tissue, causing a wound that may never heal properly [25].

Histological characteristics of osteonecrosis of the jaw include necrotic bone with bacterial colonies and granulation tissue [26] as well as decreased vascularization and number of osteoblasts [20]. Some biopsy specimens showed fungal and bacterial colonies. In malignancy patients treated with BPs, such lesions occur irrespective of existence of jaw metastases [26].

## 5. Risk Factors 

The exact mechanism leading to BRONJ is unknown. However, risk factors to develop this condition may be divided into three: risk factors related to drug intake, local risk factors, and systemic risk factors [7]. The AAOMS, in 2009, also mentioned anatomic traits (torus palatinus and mandibular, the mylohyoid line), advanced age, being of Caucasian descent, and other genetic specificities as additional risk factors. Despite being low, the risk of developing BRONJ increases when BP use is longer than three years, and such time is reduced for patients on chronic corticosteroids [9].

## 6. Prevention

Before treatment with intravenous BPs, a patient should undergo thorough intraoral examination followed by comprehensive dental treatment. In addition, optimal periodontal health should be regained if not present. There seems to be no contraindications for elective oral surgery in patients on oral BPs without signs of bone exposure and less than three years of drug usage. When the therapy is shorter than three years and combined with corticosteroids, the clinician should consider a “drug holiday” of three months before elective oral surgery, extended to the following three months whenever the patient's systemic conditions allow. Such considerations should also be taken if the use of BPs is longer than three years regardless of concomitant use of steroids [9].

## 7. Treatment 

To date, treatment option for patients with BRONJ is limited and predominantly palliative, aiming at relieving the main signs and symptoms [7]. Marx et al. [7] recommend that treatment should eliminate and control pain, as well as preventing progression of bone exposure through antibiotics therapy and mouthwash with 0.12% chlorhexidine. They also state that conservative surgical treatments are preferential, aiming at nonexposure of necrotic bone boundaries. AAOMS in 2009 recommended the removal of well-defined bone sequestrations, as well as the removal and/or relining of bone necrosis areas which are a constant source of irritation to soft tissues [9].

Montebugnoli et al. [27] also recommended the management of osteonecrosis with nonsurgical protocol. These authors conducted a study dividing patients into two groups, one treated with surgery and the other treated with antibiotics. Data analysis showed there was no statistically significant difference between outcomes for the two groups.

Curi et al. [28] reported on three clinical cases in which sequestra removal was performed and autologous platelet-rich plasma was topically applied onto the remaining defect. After six-month follow-up, complete repair of surgical site was seen, thus showing promising results.

Discontinuation of oral BPs in BRONJ patients has been associated with gradual improvement of clinical disease [29]. Discontinuation for 6–12 months may result in sequestration with spontaneous resolution after surgical debridement. Whenever systemic conditions permit, changing or stopping oral bisphosphonate treatment must be a result of an agreement between the professionals involved and the patient [9].

## 8. Discussion 

Intravenous BPs are usually considered stronger than those given orally. Therefore, use of intravenous BPs is one of the main risk factors to induce BRONJ, as evidenced by the higher estimates of BRONJ incidence (0–10%) in patients treated with IV drugs as compared to an oral therapy (<1%) [30, 31].

Among the BPs, those more likely to induce BRONJ are amino-BPs [25, 32, 33], possibly because they are stronger than alkyl-BPs. Pamidronate, alendronate, and zoledronate are 10, 100, and 1000 timer stronger than clodronate, respectively [31]. Bone necrosis is considered dose- and time-dependent due to the long half-life of BPs in the bone [25].

In patients who used BPs and did not experience osteonecrosis, preventive measures should be taken, since osteonecrosis may appear up to one decade after the start of bisphosphonate therapy. Patient should be advised to undergo thorough oral examination every 3 months [34].

If osteonecrosis develops, nonsurgical management may be beneficial and is based on antibiotic therapy. A “drug holiday” may be considered in severe cases if benefits overcome the risks of bone complications, although improvement has not been observed to date [33, 35]. Similarly, hyperbaric oxygen therapy is not effective and, therefore, not recommended [35].

## 9. Conclusions

The importance of a good anamnesis and history taking greatly helps the healthcare professional in the correct diagnosis of BRONJ lesions. Warning patients of the necessary care and the potential oral manifestations, which are often forgotten or ignored, and maintaining a professional relationship with the accompanying physician and/or oncologist are essential for the good clinical management of patients on BPs. All healthcare professionals should provide guidance for patients on BPs in the sense that good oral health should be kept by all means, since oral surgical treatment could lead to BRONJ. For those patients using BPs but who have not experienced BRONJ, preventive measures should be taken, as the condition may appear up to one decade after therapy start. Appropriate oral hygiene, along with frequent oral examination and minimally invasive dental treatment, when needed, are all clinical choices that must be adopted in order to avoid BRONJ development.

## Figures and Tables

**Table 1:** Stages of BRON-J adapted from Ruggiero et al. [17].

Stage 1	Exposed bone, asymptomatic and without evidence of inflammatory, or infectious reaction in the adjacent soft tissue

Stage 2	Exposed bone with associated pain, edema, and inflammation of the adjacent soft tissue and/or secondary infection

Stage 3	Exposed bone, with associated pain, inflammation, and infection of the adjacent soft tissue, which is hard to manage only through oral or intravenous antibiotics therapy; the presence of extraoral skin fistula secondary to osteonecrosis or a pathologic fracture is common among patients in this stage
